# Mechanical Properties and Strengthening Contributions of AISI 316 LN Austenitic Stainless Steel Grade

**DOI:** 10.3390/ma18030499

**Published:** 2025-01-22

**Authors:** Tibor Kvackaj, Jana Bidulská, Alica Fedoríková, Róbert Bidulský

**Affiliations:** 1 Bodva Industry and Innovation Cluster, Budulov 174, 04501 Moldava nad Bodvou, Slovakia; director@biic.sk; 2 Institute of Materials, Faculty of Materials, Metallurgy and Recycling, Technical University of Košice, Letná 9, 04200 Košice, Slovakia; 3 Department of Material Analysis, Research Centre Řež, Hlavní 130, 25068 Husinec, Czech Republic; alica.fedorikova@cvrez.cz; 4 Advanced Research and Innovation Hub, Budulov 174, 04501 Moldava nad Bodvou, Slovakia

**Keywords:** AISI 316 LN, ambient rolling, mechanical properties, strengthening contributions

## Abstract

The main goal of this contribution is to evaluate the mechanical properties, strengthening contributions and microstructure development of austenitic stainless steel AISI 316 LN with high nitrogen content in the states characterized as the initial state and the states after rolling with different thickness deformations. The initial state was represented by solution annealing (777 K/60 min). The deformation state was characterized by rolling thickness reductions carried out at ambient temperature (*T_A_* = 295 K) with deformations in the range ε ∈ (0; 50> [%]. Studies of microstructures, mechanical properties and strengthening contributions before and after rolling were carried out. The initial state after solution annealing was as follows: offset yield strength R_p0.2_ = 325 MPa, elongation A5 = 49% and diameter of grain size d = 214 μm. The state after ambient rolling with thickness deformation ε = 50% was as follows: R_p0.2_ = 994 MPa, A5 = 4% and d = 64 μm. The maximum contribution to strengthening after rolling processing with 50% thickness deformation was dislocations (∆ R _P0.2_DS_ = 560 MPa) followed by twins (∆ R _P0.2_DT_ =140 MPa).

## 1. Introduction

Austenitic stainless steels (SSs) are generally characterized by moderate strength, good ductility, formability, weldability and low temperatures, toughness, paramagnetic properties and good corrosion resistance [1]. SSs have wide applications in various industrial and health sectors such as, e.g., the food industry, petrochemical industry, chemical industry and medicine in the form of artificial implants, energy and nuclear power plants, casing tubes for petroleum production, hydrogen transport, marine tanks and architectural elements [2,3,4,5,6]. The moderate strength properties of austenitic SS, precisely the yield strength as an important parameter for designers, is primarily a function of the face-centered cubic (FCC) crystallographic system of austenite [2] and the ability to initiate dynamic recovery and dynamic recrystallization depending on thermo-deformation conditions [7]. To increase the basic value of the moderate yield strength, it is necessary to use other additional strengthening mechanisms such as grain boundaries, dislocations, twinning and precipitation effects. If the use of SS is considered for structural elements working at cryogenic temperatures, e.g., in a fusion reactor (ITER), the basic suitable steel grade is AISI 316 L [8]. The base components of ITER are superconducting magnets such as the 18 toroidal coils, the central solenoid, the 6 poloidal coils and the 18 correction coils (CCs) [9] and other structural parts such as plates, forgings and tubes [10]. Since AISI 316 L steel as a basic material for work in cryogenic conditions shows only a moderate value of yield strength, a new grade of AISI 316 LN steel was developed, which has considerably higher yield strength values, achieved by the use of additional strengthening mechanisms [11,12,13] such as solid solution strengthening with interstitial elements (∆R_P0.2_IS_), refinement of the final grain diameter (∆R_P0.2_GB_), dislocation strengthening (∆R_P0.2_DS_), strengthening from deformation twinning (∆R_P0.2_DT_), precipitation strengthening (∆R_P0.2_PS_) and strengthening contribution from Peierls–Nabarro stress (∆R_P0.2_PN_). The strengthening effect from a solid solution is very effective because of the small contents of interstitial elements such as nitrogen and carbon; therefore, the steel is designated as AISI 316 LN. Because carbon deteriorates the cold formability and weldability of the material, its content is limited to the level of C ≤ 0.06 mass %. On the other hand, authors [11] reported that the maximal solubility of nitrogen at steel grade AISI 316 L is 0.238 mass %.

For thick plates, the author of [14] recommends the total content of interstitial elements (C+N) in the range (C+N) ∈ <0.2;0.25> [mass%]. Authors [15] studied SS using a wide interval of nitrogen content (0.008–0.34) mass% and found that the yield strength increased from 249 MPa to 757 MPa (grain size diameter was 20 μm). The authors of [16] reported that the contribution to solid solution strengthening by interstitial elements is ∆R_P0.2, IS_ ≈ 97 MPa. Other authors [17,18,19,20] reported relationships between nitrogen content and yield strength.

In addition, micro-alloying elements such as niobium and boron can achieve tubular strengthening effects. A further increase in yield strength can be achieved by strengthening contributions from Nb, which result from suppressed recrystallisation kinetics during hot material processing with a strong effect on refining final grain size and precipitation strengthening by Nb(C, N) fine precipitates. The effective content of niobium is in the interval (0.02–0.04) mass % [21]. The strengthening contribution from the precipitation effect of Nb(C, N) precipitates can be ∆R_P0.2, Nb_Prec_ ≈ 10 MPa/0.01 mass %, and from grain boundaries, ∆R_P0.2, Nb_GB_ ≈ 25 MPa/0.01 mass %.

Also, boron is considered a very effective microalloying element, with the B content ≤ 0.0025 mass %. Boron is a typical grain boundary segregation element in steel, increasing grain boundaries’ plasticity resistance and contributing to yield strength. Boron inhibits the formation and propagation of intergranular cracks in cryogenic conditions [22,23]. The strengthening mechanism resulting from grain size refinement and derived regression equations describing the dependence between grain size yield strength and diameter were widely discussed using the Hall–Petch formula [24]. Authors [25] reported that the contribution resulting from the refinement of the grain size strengthening of steel AISI 316 LN can be ∆R_P0.2, GB_ ∈ <60;90> [MPa]. The contribution from grain refinement is very effective because it does not require an increase in the proportion of alloying elements and increases the resistance to brittle fracture at low temperatures. Classic plastic deformations realized, e.g., by controlled regimes of rolling, forging and extrusion in hot, followed by cold deformation conditions, allow a reduced final diameter of grain size from coarse grain diameter (≈100 μm) to the interval d ∈ <2;4> [μm] [26].

Subsequently, further grain size refinement options are characterized by severe plastic deformation (SPD) techniques performed at ambient and cryogenic temperatures [27,28,29]. Applications of SPD techniques have made it possible to achieve finite grain sizes at the level of ultrafine grains (UFGs) with a diameter of 500–100 nm and grains at the nanoscale level (NGs) with a diameter of d_NG_ < 100 nm. A significant strengthening effect that depends on plastic deformation is dislocation strengthening. According to the authors of [25,30,31], the contribution from dislocation strengthening lies in the interval ∆R_P0.2,Disl_ ∈ <166;363> [MPa]. The strengthening contribution from Peierls–Nabarro stress, or lattice friction stress, represents the force required to move dislocations within a plane of atoms in the unit cell. This contribution for SS can lie in the interval ∆R_P0.2, PN_ ∈ <5;60> [MPa] depending on the carbon content.

The grade AISI 316 LN steel in its basic state after annealing is characterized by a coarse-grained microstructure (≈100 μm) and low strength properties such as R_P0.2_ ≤ 300 MPa, Rm = 550–750 MPa and A5 > 35% [32]. On the other hand, AISI 316 LN SS is characterized by a good combination of ductility and excellent resistance to corrosion cracking at both low temperatures (223 K) and high temperatures (723 K). It also has better formability than steel grades AISI 304 or AISI 309 due to its lower carbon content. It can be easily formed into more complex geometry shapes from input materials, such as sheet metal or tubing, without requiring much heat treatment. Hardness ranges from approximately 70 HRC to 75 HRC depending on the chemical composition and heat treatment process used in production [33]. After applying traditional metal forming processes such as commercial cold plastic deformations, the yield strength can be increased to R_P0.2_ ≤ 600 MPa. Because the current requirements for the strength properties of AISI 316 LN steel intended for ITER components rise significantly to R_P0.2_ > 1200 MPa and KIC(J) > 200 MPa/m^2^, obtained at 4.2 K [14,17,34], it has become necessary to find a new way of increasing them. SPD techniques are one effective way to rapidly increase the yield strength without reducing the ductility [35,36,37,38,39]. These studies indicated that the yield strength of the SS with UFG or NG structures allows values in the intervals R_P0.2, UFG_∈ (1100;1500) [MPa] and R_P0.2,NG_ ∈ (1700;2200) [MPa] to be reached, which provide attractive properties for the applications that follow. Plastic deformations performed at cryogenic temperatures represent another unique technique for increasing yield strength. Classical rolling and asymmetric rolling at cryogenic temperatures (CRs) in liquid nitrogen increased the yield strength by structure refinement up to the level of UFG. Authors published the first information about the rolling of SS at cryogenic conditions in 1991 [40]. Broader applications of CR techniques for SS aimed at increasing the yield strength were only presented later, e.g., [41,42,43,44,45,46]. The plastic deformations of SS carried out at cryogenic temperatures lead to a remarkable increase in strength and hardness and a decrease in elongation. The base plastic deformation mechanisms of austenite—Fe(γ)—with a face-centered cubic (FCC) crystallographic system are ε or α’ martensitic transformation, mechanical twinning and dislocation slips [47,48]. Activating the separate mechanism of plastic deformation depends on the value of the stacking fault energy (SFE) of austenite [48,49]. The authors of [50,51] report that as the SFE value of austenite increases, the plastic deformation mechanisms of austenitic steel change from martensitic transformation to the mechanical twinning mode and subsequently to a dislocation slip. The authors of [11,52] mentioned that planar slips, stacking faults and twinning are deformation mechanisms in deformed structures of AISI 316 LN SS containing higher nitrogen content, which are factors for the significant improvement in the yield strength.

Structure refinement causes martensite nucleation to decrease and raises the propensity to mechanical twinning. Both strain-induced martensite and deformation twinning contribute to strain hardening. In general, there are the following two types of transformation mechanisms of AISI 316 LN from austenite to martensite, as proposed by the authors of [43,53,54]:

(i) When the stacking fault energy of the base metal is (γ_SFE_ < 18 mJ/m^2^), the transformation of martensite follows the route of (γ_fcc_ -austenite → ε_hcp_ -martensite → α′bcc-martensite). (ii) When the stacking fault energy of the base metal is (γ_SFE_ > 18 mJ/m^2^), the route is (γ_fcc_-austenite → twin → α′bcc-martensite). Other authors [47] specify that if the SFE value is low (γ_SFE_ < 20 mJ/m^2^), the phase transformation from the austenitic phase to the martensitic phase (ε- or α’-martensitic transformations) is preferred. If the SFE has an intermediate value lying in the interval (γ_SFE_ ∈ <20;45> [mJ/m^2^]), the twinning is a deformation mechanism that raises the work hardening rate without transformation to α′-martensite. If the SFE value is high (γ_SFE_ > 45 mJ/m^2^), the deformation process is mainly controlled by dislocation slip. Also, the analysis of the authors of [55] shows that nitrogen content of approximately 0.1 mass% has a braking effect on the formation of α′martensite. The authors of [35] mention that the ability to nucleate martensite decreases with a decrease in grain size, and the transformation of austenite to strain-induced martensite is largely suppressed. The stability of austenite increases with a reduction in grain size. Table 1 [56,57,58,59,60] gives the derived equations describing the SFE calculation depending on SS’s chemical composition.

Analyzing the given regression equations strongly affects the rise in SFE: carbon, followed by nickel. On the other hand, silicon has the highest decreasing effect on SFE, followed by nitrogen. The other discussed chemical elements show a weak influence on SFE.

The precipitation effect in the 316 grade steel depends on the nitrogen content [61]. When a small percentage of nitrogen is present in the steel, the formation of M_23_C_6_ can be expected. The authors of [61] mention that the most favorable sites for M_23_C_6_ carbide precipitation are grain boundaries, followed by incoherent twin boundaries, coherent twin boundaries and finally, the dislocation formation within the grains. An interesting finding by the author of [61] is that many carbides can form after only annealing for a few minutes between 923 and 1023 K.

The present paper focuses on the investigation of the influence of plastic deformations realized by rolling at ambient temperatures with the aim of the maximization of mechanical properties and the description of observations of the strengthening contribution of AISI 316 LN austenitic stainless steel as a material with an FCC crystallographic lattice on the base of microstructures. The fundamental contribution of the article results from research on increasing yield strength values with the identification of the effectiveness of the strengthening contributions of the bulk AISI 316 LN material investigated under various cold plastic deformation conditions, which have not been studied before, as well as the study of the mechanisms of plastic deformation.

## 2. Materials and Experimental Procedure

For experimental studies, ultra-low-carbon (ULC) austenitic grade AISI 316 LN SS, which contained nitrogen, niobium and boron, was used, with the chemical composition given in Table 2.

**Figure 1:**
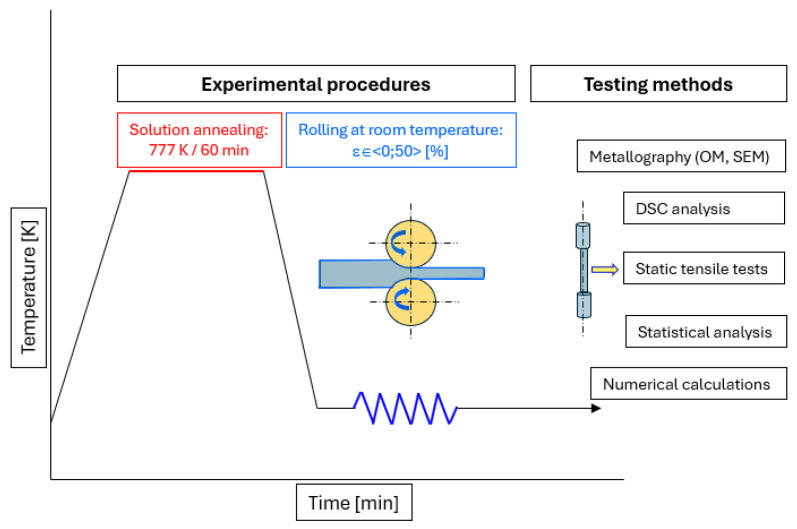
Experimental procedures and testing methods for study of material AISI 316 LN.

From the middle part of a hot forged ingot, the rectangular samples for rolling which were h_0_xb_0_xl_0_ = 15 × 40 × 75 mm in size were cut, which were subsequently treated by solid solution annealing at 777 K for 60 min followed by being quickly cooled with pressure air to ambient temperature. The rolling process was carried out at ambient temperatures (295 K) in a duo rolling mill with a rolling diameter of 210 mm and a 1 m/min rolling speed with a friction coefficient f = 0.28 resulting from dry rolling by steel rolls. Samples were cold rolled by a 10% reduction per pass with a total thickness reduction of 10%, 30% and 50%. The experimental scheme is given in Figure 1.

After experimental rolling, metallographic, DSC analysis and static tensile test samples were taken. The microstructures were analyzed using light optical microscopy (LOM) and Scanning Electron Microscopy (SEM). The orientation of the specimen planes for LOM and SEM analyses was to the thickness of the rolled material parallel to the rolling direction. Differential scanning calorimetry (DSC) analysis was conducted at a 30 K/min heating rate under a gas nitrogen atmosphere. Static tensile tests were performed at ambient temperature (295 K) on round specimens, which were machined from rolled samples with a diameter and length of measurement part d = 4 mm, l = 22 mm (ASTM E8M standard), with a displacement rate of 0.5 mm/min. Each series of static tensile tests consisted of three specimens with axes of symmetry oriented parallel to the rolling direction.

## 3. Results and Analysis

### 3.1. Microstructure Analysis

#### 3.1.1. Initial State After Solution Annealing

**Figure 2:**
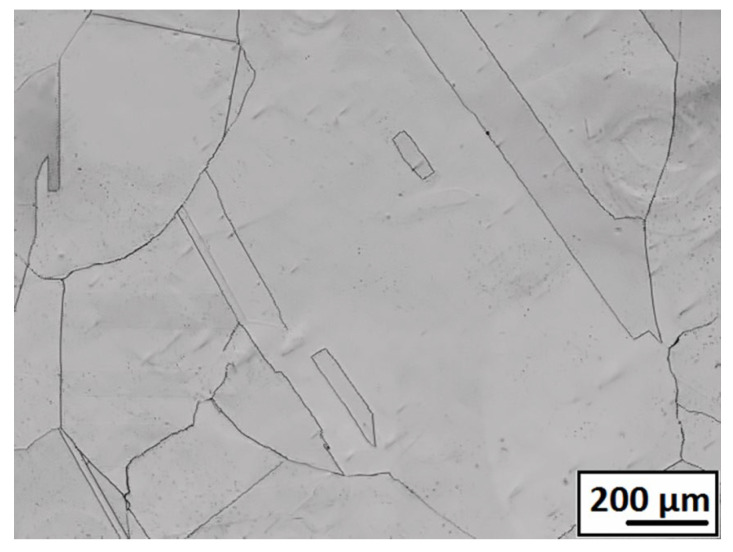
Initial microstructure after SA studied by LOM.

Figure 2 shows the micrographs from microstructure observations using LOM after solution annealing before rolling. Coarse polyhedric austenite grains with a grain size diameter of 214 μm and a small occurrence of annealed twins were observed.

#### 3.1.2. Study of Microstructures After Sample Processing at Ambient Temperatures

**Figure 3:**
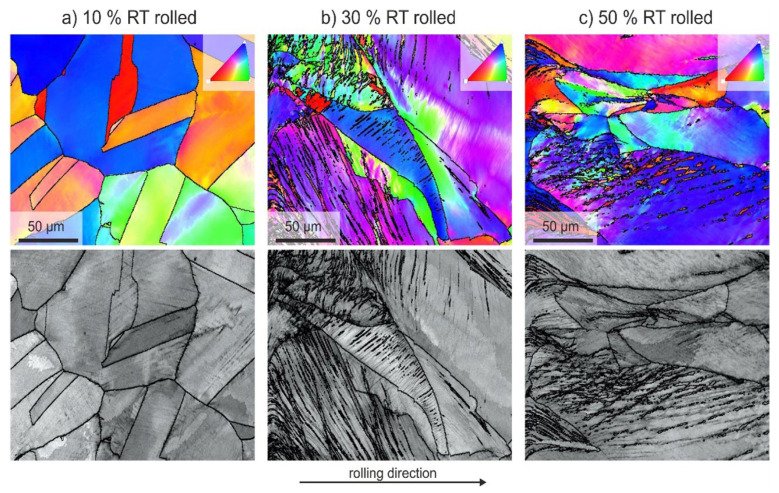
SEM—orientation mapping (upper figures) and image quality maps (lower figures) after rolling at room temperature (RT), 295 K and different total deformation (the color code corresponds to the inverse pole figure of the z-direction. The maps are of the same size and observed using a step size of 1 μm). [(**a**) Rolling deformation of the material thickness ε = 10%, (**b**) rolling deformation of the material thickness ε = 30%, (**c**) rolling deformation of the material thickness ε = 50%].

Figure 3 shows the orientation mapping (upper figures) and image quality maps of different deformed samples by 10%, 30% and 50% deformations. The color code corresponds to the inverse pole figure, and the IPF of the sample surface is normal.

The 10% deformation at 295 K caused former grain boundaries; annealing twins and grains with original grain sizes were still visible, as is shown in Figure 3a. In Figure 3b, former grain boundaries, deformation twins and deformed grains with gradual orientation in the rolling direction are observed. Inhomogeneous gray contrast in single grains resulted from a stronger increasing number of dislocations. After 50% deformation, the former grain boundaries and grains with the original size were only slightly visible, as shown in Figure 3c. The inhomogeneous gray contrast indicates an increased formation of dislocations in individual grains. A high number of deformation twins and low-angle grain boundaries were also observed. More grains were elongated in the rolling direction (pancake-like shape), and a change in grain orientation to <111> (blue color in the color code) was observed.

**Figure 4:**
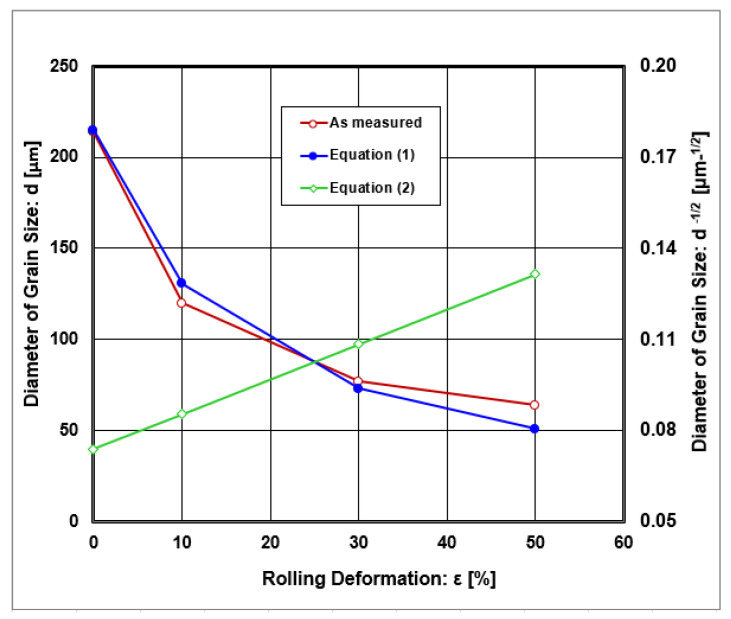
Graphical dependences on the diameter of grain size on rolling deformations.

Figure 4 shows that the diameter of austenite grains depended on rolling reduction. Graphical dependencies show that with increasing thickness deformation, the diameter of grain size decreased. The grain size diameter had a decreasing tendency with plastic deformations but remained in the region of coarse grain microstructures. These changes can be described by the curves derived by linear regression analysis with Equations (1) and (2) with index correlations R^2^ = 0.989 and R^2^ = 0.98, respectively.

(1) $$d=3330/\left(\varepsilon +15.5\right)$$

(2) $$d^{-1/2}=0.074+2810/\left(\varepsilon +1.4\right)$$

where the following definitions apply:

d [μm]—grain diameter;
ε [%]—relative thickness reduction.

### 3.2. Isothermal DSC Analysis and Analysis of Dislocation Density

**Figure 5:**
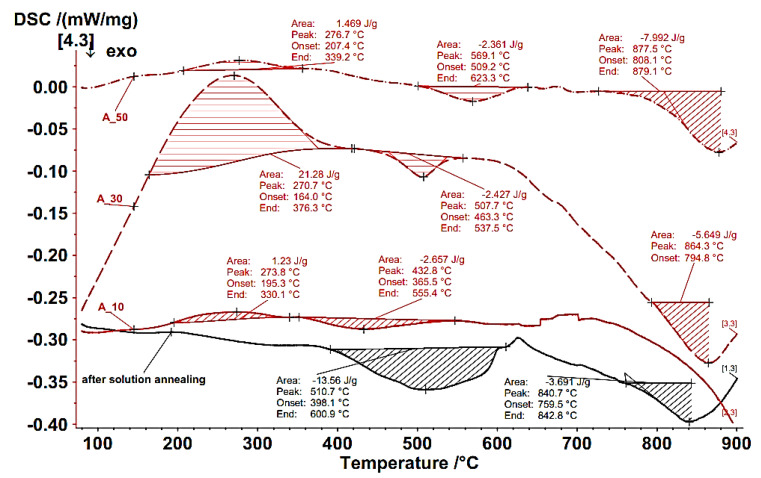
DSC thermograms of sample processing at 295 K after SA and thickness deformations ε = 10%, 30%, 50%. (Note: Temperature is given from the device as data in the physical unit [°C].)

DSC thermograms of samples processed by rolling at 295 K are given in Figure 5.

The analysis of DSC curves after material processing by rolling is given in Table 3.

Figure 5 shows the sample treated by solution annealing, characterized by two exothermic peaks and temperatures of 511 °C and 841 °C.

(i) According to [62,63], the first exothermic peak is defined as the reverse transformation of α′-martensite to γ-austenite, which lies within the temperature interval TSA, α′ → γ <467;673> [°C]. From the real DSC, the diagram shows that this transformation was observed at the temperature interval TSA, α′ → γ <398;601> [°C].
(ii) The second exothermic peak resulted from the DSC measurement lying in the temperature interval T_M23C6_<759;843> [°C] and, according to the authors of [64,65,66], is related to the formation of M_23_C_6_ carbide. The carbon solubility equilibrium state in the M_23_C_6_ carbide of AISI 316 austenitic stainless steel was described by the authors of [67] by the following formula:

(3) $$\log\left(C\left[ppm\right]\right)=7.771-\left(6272/T\right)$$

The solubility temperature of the carbon can be expressed by Equation (3) as follows:

(4) $$T\left[{}^{\circ}C\right]=\frac{6272}{7.771-\log(C\left[ppm\right])}-273=\frac{6272}{7.771-\log(600)}-273=983{}^{\circ}C$$

Based on the previous calculation, it can be supposed that the dissolution of all M_23_C_6_ carbide will be finished at a temperature of 983 °C. On the other hand, authors [61] reported that the temperature range of the formation of M_23_C_6_ carbide is in intervals at 600–950 °C and the authors of [61] mentioned that this temperature interval is 650–750 °C. The measured DSC data for the second peak showed that the M_23_C_6_ carbide formation temperature lies in the interval T_M23C6_ ∈ <759;843> [°C], which falls within the interval set by the authors of [61]. It is possible to assume that these carbides are active in raising the strength properties of the observed material.

Three peaks characterized the samples processed by rolling with different deformations at 295 K. The first peak showed an endothermic thermal effect, and the others showed exothermic thermal effects. The peaks resulting from endothermic thermal effects, stored energies and dislocation densities have the following values:

- Deformation 10%: T_A10_peak1_ ∈ <195;330> [°C] with stored energy:
  ∆H_A10_peak1_ = 1.23 J/g and calculated dislocation density: ρ_A10_peak1_ = 1.74 × 10^+15^ m^−2^.
- Deformation 30%: T_A30_peak1_ ∈ <164;376> [°C] with stored energy:
  ∆H_A30_peak1_ = 21.28 J/g and calculated dislocation density: ρ_A30_peak1_ = 6.8 × 10^+15^ m^−2^.
- Deformation 50%: T_A50_peak1_ ∈ <207;339> [°C] with stored energy:
  ∆H_A50_peak1_ = 1.469J/g and calculated dislocation density: ρ_A50_peak1_ = 5.17 × 10^+15^ m^−2^.

The first endothermic peaks are characterized by the development of stored energy, which is the source of all the strengthening effects that occur during deformation and are derived from the point defects and dislocations generated during deformation [68]. The authors of [68] described the relationship between stored energy and dislocation density by the following formula:

(5) $$∆H=c_{2}\cdot \rho \cdot G\cdot b^{2}$$

The expression of the dislocation density from Equation (5) has a form, as follows:

(6) $$\rho =\frac{∆H}{c_{2}\cdot G\cdot b^{2}}$$

where the following definitions apply:

c_2_ [-]—numerical factor;
G = 75 [GPa]—shear modulus;
b = 0.25597 [nm]—Burger’s vector.

**Figure 6:**
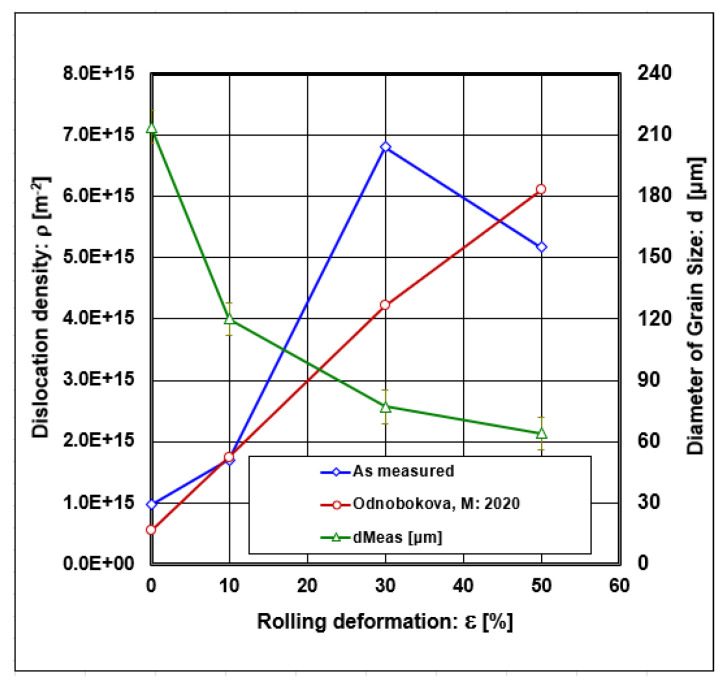
Dislocation density was calculated using equations (Equations (6) and (7)) and grain size diameter vs. rolling deformations [69].

The development of dislocation density after material rolling depends on deformations, which are given in Figure 6.

Graphical dependence results in a variable change in dislocation density. The dislocation density increases with 30% deformation, where the maximum is reached, and then decreases weakly. The decrease in dislocation density after 30% deformation is caused by a reduction in the proportion of dislocation slip and a rise in deformation twinning.

The authors of [69] proposed the calculation of the dislocation density depending on the diameter of grain size according to the following formula:

ρ = (C_0_/d) (7)

where the following definition applies:

C_0_ [-]—constant.

Graphical dependencies of dislocation density calculated according to Equations (6) and (7) are shown in Figure 6. These dependencies show that the curve calculated using Equation (6) shows one local extremum, while the curve calculated by Equation (7) tends to be a monotonically increasing function. The graphical dependencies show that the simple Equation (7) reasonably describes the dislocation density evolution at the first and last point but does not cover the local maximum.

**Figure 7:**
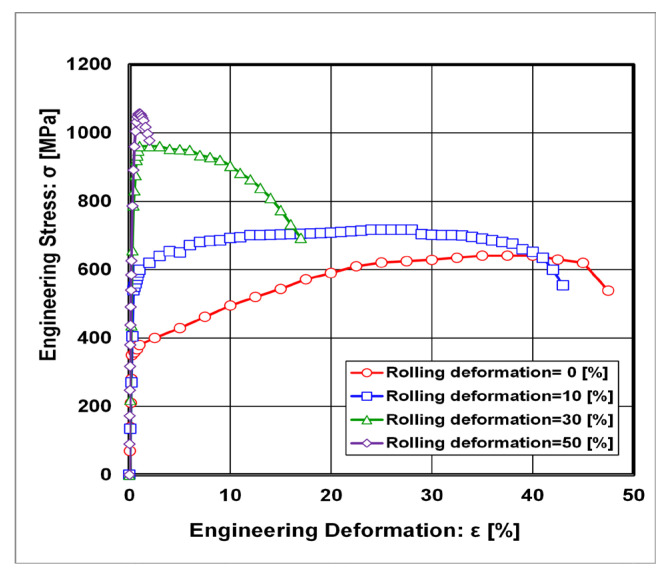
Stress–strain curves after static tensile tests at 295 K depending on different thickness rolling deformations.

**Figure 8:**
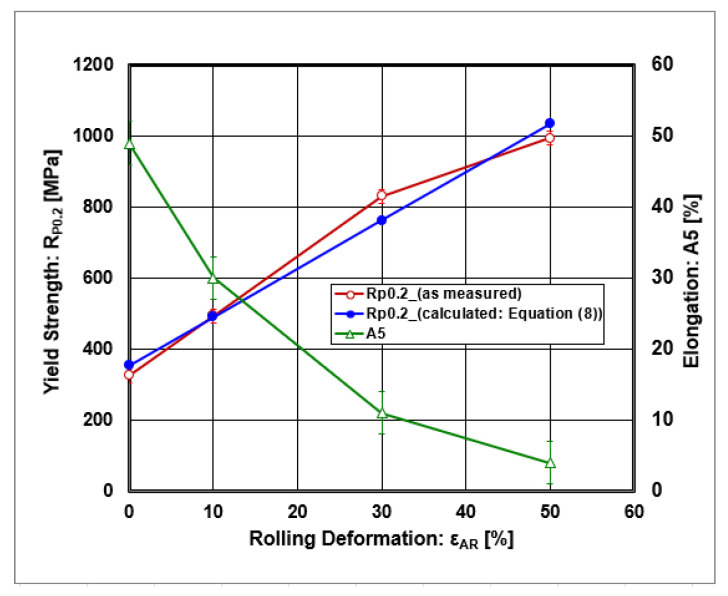
The dependences of offset yield strength and elongation on rolling deformations.

If the curves from static tensile tests were evaluated as the values of offset yield strength R_P0.2_ and elongation A_5_ depending on rolling deformations, their graphic course is given in Figure 7 and Figure 8.

The approximation equation describing the dependence of the offset yield strength on rolling deformations has the following form with index correlations R^2^ = 0.987:

(8) $$R_{P0.2}=354+13.6\cdot \varepsilon$$

where the following definitions apply:

R_P0.2_ [MPa]—the offset yield strength;
ε [%]—thickness rolling reduction.

**Figure 9:**
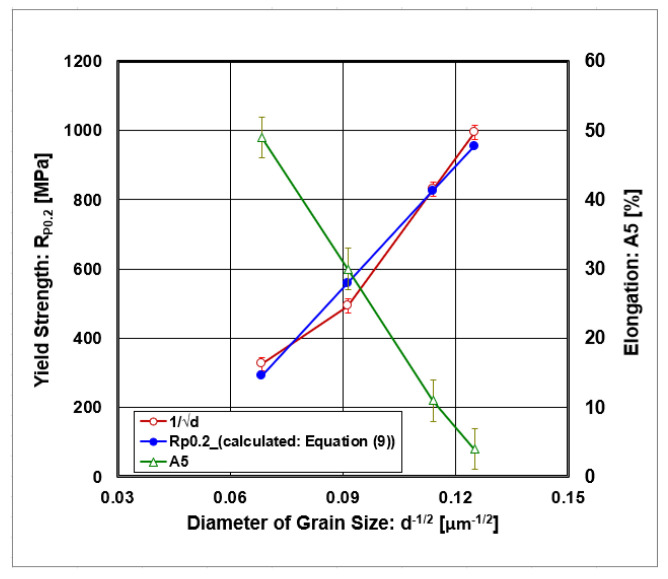
The dependences of offset yield strength and elongation on rolling deformations.

From dependencies, there is a linear increase in the offset yield strength and a decrease in elongation on the dependence at thickness rolling reduction. The dependence of the offset yield strength on the inverted diameter of grain size is graphically given in Figure 9 and numerically described by the following regression equation (R^2^ = 0.989):

(9) $$R_{P0.2}=R_{0}+k_{Y}\cdot d^{-1/2}=-511+11720\cdot d^{-1/2}$$

where the following definitions apply:

R_0_ [MPa]—yield stress in single crystal (friction stress);
k_y_ [MPa.μm^−1/2^]—Hall–Petch coefficient describing the grain boundary strengthening;
d^−1/2^ [μm^−1/2^]—the inverse square root of the grain size diameter.

The authors of [70] also described a negative value of frictional stress (R_0_). The increase in this value is ascribed to the resistance to the movement of dislocations in the grain interior. They also declared that the Hall–Petch coefficient (k_y_) is significantly higher for refined grains than for coarse grains.

### 3.3. Mechanisms of Plastic Deformation

Based on the measured data, it can be concluded that the increase in deformation rapidly increases the dislocation density. This assumption should be valid when only one separate deformation mechanism is active. Since the dislocation density does not show a monotonically increasing curve depending on increasing deformation, it can be assumed that under certain conditions, another parallel deformation mechanism can be activated, which reduces the dislocation density. According to the authors of [43,53,54], stainless steels with SFE > 18 mJ/m^2^ as an additional deformation mechanism will act in deformation twinning. Table 4 calculates SFE according to the authors of [56,57,58,59,60].

From Table 4, the interval of calculated values is γ_SFE_ ∈ <24;41> [mJ/m^2^], which classifies the observed FCC material between the materials with low to medium γ_SFE_. The authors of [47] mention that if the SFE values lie in the interval γ_SFE_ ∈ <20;45> [mJ/m^2^], twinning is one of the deformation mechanisms that raise the strain hardening. If the SFE value is high (γ_SFE_ > 45 mJ/m^2^), the deformation process is mainly controlled by dislocation slip. Regarding the strain hardening effect from α ′ martensite in the studied SS (nitrogen content of 0.13 wt.% and an SFE value lying in the interval γ_SFE_ ∈ <20;45> [mJ/m^2^]), it is realistically assumed that this effect will not act to increase the yield strength value. The authors of [69] state that twinning occurs if SFE lies in the interval γ_SFE_ ∈ <18;45> [mJ/m^2^], which also includes the calculated values in Table 4.

The authors of [71,72] described the shear stress for deformation twinning as depending on grain size by the following formula:

(10) $$\tau_{DT,d}=\frac{G\cdot a}{2\cdot \sqrt{6}\cdot \pi \cdot d\cdot \sin(\alpha )}\cdot \ln(\frac{\sqrt{2}\cdot d}{a})=\frac{G\cdot a}{2\cdot \sqrt{6}\cdot \pi \cdot 0.4462\cdot d}\cdot \ln(\frac{\sqrt{2}\cdot d}{a})=\frac{G\cdot a}{6.86\cdot d}\cdot \ln(\frac{\sqrt{2}\cdot d}{a})$$

where the following definitions apply:

a = 0.3619 [nm]—is the lattice parameter;
d [nm]—diameter of grain size.

The authors of [73] defined the minimal value of shear stress for twinning nucleation by the following linear formula:

(11) $$\tau_{DT,\gamma_{min}}=\frac{\left(5.69-2.02\cdot \nu \right)\cdot \gamma_{SFE}}{2\cdot a}$$

where ν = 0.294 [-]—Poisson’s ratio.

The authors of [74] described the maximal critical shear stress for deformation twinning by the following linear formula:

(12) $$\tau_{DT,\gamma_{SFE},max}=2\cdot \frac{\gamma_{SFE}}{b_{p}}$$

The recalculation of shear stress to normal stress can be performed according to the following von Mises formula:

(13) $${\sigma_{DT}=\sqrt{3}\cdot \tau }_{DT}$$

**Figure 10:**
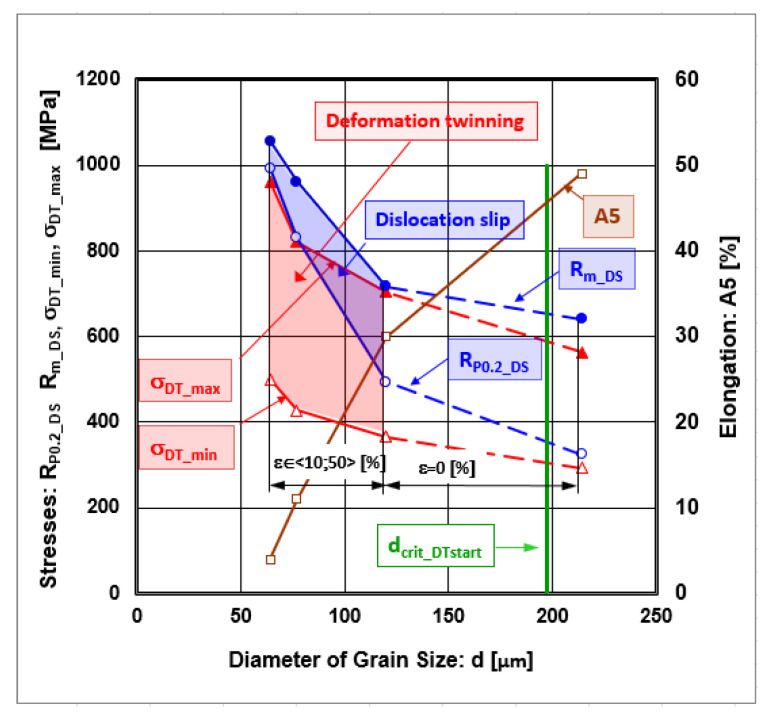
Dependence of the stresses required to trigger the deformation mechanisms (dislocation slip, deformation twinning) and elongation on the grain size diameter.

Curves describing the development of normal stresses and strength properties depending on the grain size diameter are given in Figure 10. In addition, it was necessary to define the plastic deformation zone to understand when the deformation mechanism occurs through deformation twinning or dislocation slip. The zone of plastic deformation, named the “zone of plastic stability”, lies between the starting point offset yield strength and the finishing point tensile strength, i.e., in the interval <R_P0.2_; Rm> [MPa]. The zone of stable plastic deformation is where deformation slips occur. Suppose the values describing the minimum and maximum shear stresses are calculated using Equations (11) and (12) and are subsequently recalculated, according to the von Mises formula, to the normal stresses. In that case, the space between the minimum and maximum values of the normal stresses creates a zone for deformation twinning. The areas of normal stresses for deformation twinning and deformation slip overlap, resulting in deformation twinning as an active mechanism from the beginning of deformation, and its role rises with the increasing deformation. When the deformation is ε = 10% (d = 120 μm), both mechanisms of plastic deformation take place in parallel. Also, the slope of the elongation line changes, which means there is a change in plastic deformation mechanisms. If the deformation is ε ≥ 30% (d ≤ 77 μm), the normal stress required for twinning is lower than that needed for dislocation slip, and deformation twinning becomes more dominant.

The authors of [75] described the critical diameter of grain size at which twinning begins with the following formula:

(14) $$d_{crit_{\_DT}\_start}=\frac{2\cdot G\cdot b_{p}}{\gamma_{SFE}}\cdot \left(n\cdot b-b_{p}\right)$$

where n [-]—stress concentration factor (n = 2).

According to Equation (14), the calculated value for the start of d_crit_DT_start_ = 197 μm. Decreasing the grain size diameter minimizes the role of dislocation slip, accompanied by deformation twinning. Figure 6 shows that the dislocation density after deformation >30% decreases, which confirms the decreasing role of dislocation slip, as shown in Figure 10.

Grain refinement is an effective method of improving material strength and is widely utilized. Grain boundaries represent obstacles to the movement of dislocations and twins and thus lead to a strengthening effect. The grain size effect on deformation twinning and dislocation slip is inversely proportional to the grain size according to Equations (22) and (27). Suppose we substitute Equation (7) describing the dislocation density into the Taylor Equation (26) for the strengthening contribution. In that case, we obtain a formula expressing the dependence of the strengthening contribution from the dislocation density on the inverse character of the grain size. Comparing the Hall–Petch formulas describing the strengthening contribution from grain boundaries using Equation (22) and the strengthening contribution from deformation twinning using Equation (27), it can be seen that the effect of grain size on deformation twinning is stronger than that on dislocation slip because the inequality given by Equation (28) is valid. Other authors confirmed the validity of the inequality described by Equation (28) [76]. The dependencies show that the effect of grain size on deformation twinning is stronger than that on dislocation slip [77,78]. The authors of [77,78] studied a Mg alloy and found a critical grain size of 2.7 μm when one deformation mechanism dominated. Based on our study and Figure 10, it can be confirmed that there is a critical grain size for the AISI 316 LN material, with a value of 77 μm, reached by a deformation of 30%. When the deformation exceeds 30%, deformation twinning becomes the dominant mechanism. When the deformation is less than 30%, both mechanisms occur simultaneously.

### 3.4. Strengthening Contributions to the Yield Strength

The final level of the offset yield strength (R_P0.2_) is based on the additive strengthening effects of the partial strengthening contributions, described by the following formula:

R_P0.2_ = ∆ R_P0.2_PN_ + ∆ R_P0.2_IS_ + ∆ R_P0.2_SS_ + ∆ R_P0.2_GB_ + ∆ R_P0.2_DS_ + ∆ R_P0.2_DT_ + ∆ R_P0.2_PR_ (15)

where the following definitions apply:

∆ R_P0.2_PN_ [MPa]—strengthening contribution from Peierls–Nabarro stress;
∆ R_P0.2_IS_ [MPa]—strengthening contribution of the solid solution from interstitial elements;
∆ R_P0.2_SE_ [MPa]—strengthening contribution of the solid solution from substitution elements;
∆ R_P0.2_GB_ [MPa]—strengthening contribution from grain boundaries (Hall–Petch);
∆ R_P0.2_DS_ [MPa]—strengthening contribution from dislocation;
∆ R_P0.2_DT_ [MPa]—strengthening contribution from deformation twinning;
∆ R_P0.2_PR_ [MPa]—strengthening contribution from precipitates.

(a) The strengthening contribution from the Peierls–Nabarro stress was studied by several authors, e.g., [46,79,80] who, for materials with an FCC crystal lattice, determined its value to lie in the interval ∆ R_P0.2_PN_ ∈ <112;123> [MPa].
(b) The author of [81] described the strengthening contribution of the solid solution by interstitial elements and proposed the following formula:
  ∆ R_P0.2_IS_ = 354.C + 493.N = 354.0.06 + 493.0.13 = 78 MPa (16)
(c) The strengthening contribution of the solid solution by substitution elements can be calculated according to the following formulas:
  (i) Formula according to the author of [81]:
    ∆R_P0.2_SE_ = 20.Si + 3.7.Cr + 14.5.Mo + 18.5.V + 40.Nb + 26.Ti = 109 MPa (17)
  (ii) Formula according to the authors of [82]:
    ∆R_P0.2_SE_ = 1.Cr + 5.Ni + 19.4.Mo + 6.3.P + 8.7.Si −1.5.Mn = 126 MPa (18)

From the previous calculations, the strengthening contribution of the solid solution by substitution elements lies in the interval ∆R_P0.2_SE_ ∈ <109;126> [MPa].

(d) In the calculation of the cumulative effect of strengthening contribution from the solid solution (∆R_P0.2_SS_ = ∆R_P0.2_IS_ + ∆R_P0.2_SE_), the authors of [83,84] described the following formula:

∆R_P0.2_SS_ = 496. N + 356.5. C + 20.1. Si + 3.7. Cr + 14.6.Mo = 193 MPa (19)

The cumulative effect of the solid solution strengthening contribution given by the sums of Equation (17) and Equations (18) and (19), which are as follows:

∆R_P0.2_SS_ = ∆R_P0.2_IS_Equation (17)_ + ∆R_P0.2_SE___Equation (18)_ = 78 + 109 = 187 MPa (20)

∆R_P0.2_SS_ = ∆R_P0.2_IS___Equation (17)_ + ∆R_P0.2_SE___Equation (19)_ = 78 + 126 = 204 MPa (21)

lying in the interval ∆R_P0.2_SS_ ∈ <187;204> [MPa].

(e) The strengthening contribution from grain boundaries is described by the Hall–Petch formula as follows:

∆ R_P0.2_GB_ = R_0_ + k_y_GB_. d^−1/2^ MPa (22)

where the following definitions apply:

R_0_ [MPa]—intrinsic strength, or the friction stress of the γ-iron lattice at room temperature, defined by the Peierls–Nabarro stress given by the authors of [82], as follows:

R_0_ = 2·10^−4^·G = 2·10^−4^·75·10^3^ = 15 MPa (23)

k_y_GB_ [MPa.μm^1/2^]—grain boundary strengthening coefficient.

The analysis of the grain boundary strengthening coefficients depending on the chemical composition is given in Table 5.

The previous analysis shows that the grain boundary strengthening coefficient lies in the interval k_y_GB_ ∈ <300;500> MPa.μm^1/2^ and depends on the chemical composition.

The chemical composition used by the authors of [87] is very similar to the investigated composition, so the value k_y_GB_ = 500 MPa.μm^1/2^ can be accepted. The strengthening contribution from the grain boundaries for the studied steel grade, depending on the diameter of grain size, can be calculated as follows:

- For the diameter of grain size d = 64 μm:

∆ R_P0.2_GB_max_ = R_0_ + k_y_GB_. d^−1/2^= 15 + 500.0.13 = 80 MPa (24)

- For the diameter of grain size d = 214 μm:

∆ R_P0.2_GB_min_ = R_0_ + k_y_GB_. d^−1/2^= 15 + 500.0.07 = 50 MPa (25)

**Figure 11:**
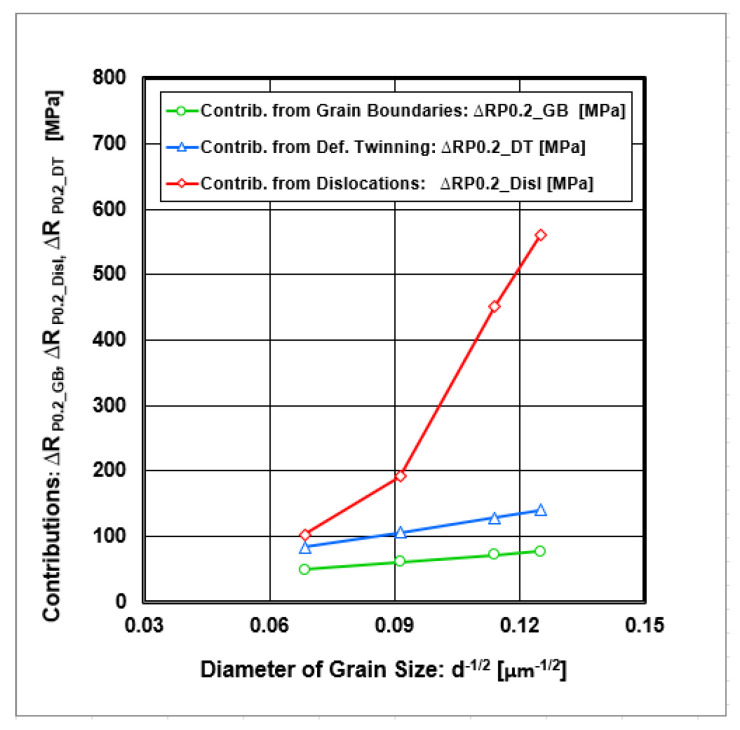
The strengthening contributions dependent on the inverse square root of the grain size diameter.

The strengthening contribution from the grain boundaries lies in the interval ∆R_P0.2_GB_ ∈ <50;80> [MPa]. The graphical dependence of the discussed strengthening contributions as a function of the grain size diameter (d^−1/2^) is shown in Figure 11.

(f) The strengthening contribution from dislocation density

The strengthening contribution from dislocations based on the Taylor equation was described, e.g., by the authors of [88,89,90], by the following formula:

(26) $$\;\;∆R_{P0.2\_DS}=\alpha \cdot G\cdot b\cdot \rho^{0.5}$$

where the following definitions apply:

α [-]—dislocation strengthening coefficient α ∈ <0.23; 0.3> [91];
G = 75 [GPa]—shear modulus;
b = 0.2559 [nm]—Burgers vector of perfect dislocation;
ρ [m^−2^]—dislocation density.

The graphical dependence of the strengthening contribution from dislocations depends on the diameter of grain size (d^−1/2^), which is shown in Figure 11, which results in the contribution lying in the interval ∆ R_P0.2_Disl_ ∈ <103; 560> [MPa].

(g) The strengthening contribution from deformation twinning

Several authors have described the strengthening contributions from deformation twinning, which are based on the following calculation approaches:

(i) Calculation based on the Taylor formula [92];
(ii) Calculation based on the volume fraction of twins [89];
(iii) Calculation based on the Hall–Petch formula [71].

It can be assumed that for the material in the undeformed state, the main parameters affecting the twinning stress are the diameter of grain size and grain orientation. For the deformed state, the deformation twinning strongly depends on the grain diameter; therefore, the Hall–Petch equation will be accepted for the calculation of the strengthening contribution from deformation twinning.

The calculation, which is based on the Hall–Petch formula, uses the following relationship:

∆ R_P0.2_DT_ = R_0_ + k_y_DT_. d^−1/2^ (27)

where the following definitions apply:

k_y_DT_ [MPa.μm^1/2^]—twin boundary strengthening coefficient.

The authors of [76] showed that between the Hall–Petch coefficients, the following inequality holds:

k_y_DT_ > k_y_DS_ (28)

and a material with an FCC crystallographic lattice is valid in the following relation:

k_y_DT_ ≈ 2. k_y_DS_ = 2.500 = 1000 MPa.μm^1/2^ (29)

The graphical dependence of the strengthening contribution from twinning depends on the diameter of grain size (d^−1/2^), which is shown in Figure 11, from which the contribution lies in the interval ∆ R_P0.2_DT_ ∈ <83; 140> [MPa].

(h) The strengthening contribution from precipitates

The experimental processing of the material was carried out in a sequence of operations: solution annealing at 777 K/60 min, cooling to ambient temperature and plastic deformation by cold rolling at an ambient temperature of 295 K without any post-deformation annealing. The experimental schedule results show that any strengthening contribution from precipitates cannot be expected.

**Figure 12:**
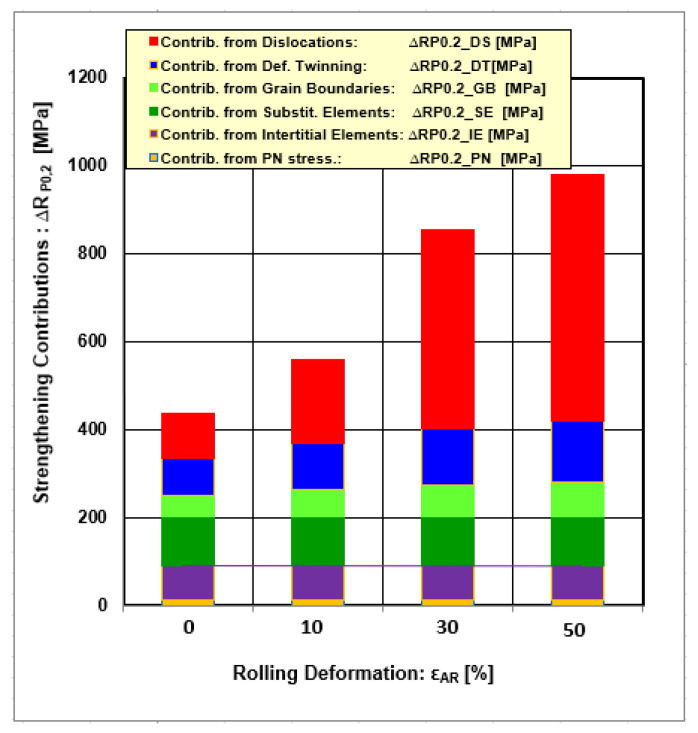
Comparison of different strengthening contributions depending on rolling deformations.

Table 6 summarizes all strengthening contributions with respect to their minimal and maximal values depending on deformations (min = 0% and max = 50%), while Figure 12 gives the graphic course valid for the whole deformation interval.

Numerical and graphical data show that there are two groups of strengthening contributions, as follows:

(i) Contributions independent from plastic deformation conditions—passive options (∆R_P0.2_PN_, ∆R_P0.2_IS_, ∆R_P0.2_SE_);
(ii) Contributions dependent on plastic deformation conditions—active options (∆R_P0.2_GB_, ∆R_P0.2_DS_, ∆R_P0.2_DT_).

The first group of strengthening contributions is dominantly dependent on chemical composition. Increasing the final yield strength depends only on the alloying elements’ content, meaning these contributions are not changeable for a given chemical composition.

The second group’s strengthening contributions depend on plastic deformations and, therefore, can be changed. The strongest effect shows a strengthening contribution resulting from the changes at dislocation density, followed by a strengthening contribution from twinning and grain size refinement. The mentioned contributions can be increased by additional processing conditions, such as plastic deformations realized as asymmetric rolling or deformations at cryogenic temperatures or combination asymmetric rolling at cryogenic temperatures [28,44,46,93,94,95,96]. Severe plastic deformation (SPD) techniques such as equal channel angular pressing (ECAP) [97,98,99,100] and equal channel angular rolling (ECAR) [27,85,101,102] are also effective methods for grain size refinement. Moreover, the last decade has shown positive responses from additive manufacturing applications, such as new concepts for end users [16,38,94,103,104,105,106].

A comparison of the strength properties reported in Table 7 showed that the current yield strength resulting from cold rolling with a thickness deformation of 50% without any annealing procedure was very close to the yield strength obtained with UFG structures using SPD techniques.

The comparison of the strength properties reported in Table 7 shows that the current yield strength resulting from cold rolling with a thickness deformation of 50% without any annealing procedure was very close to the yield strength obtained with UFG structures using SPD techniques.

## 4. Conclusions

The reduction in the carbon content to the ULC level was carried out to achieve the good weldability and cold formability of the material since carbon shows a strong negative effect on the carbon equivalent and formability.

The designation AISI 316 LN means that the basic chemical composition was supplemented with the interstitial element nitrogen to increase the yield strength caused by the low content of the interstitial element carbon.

Alloying with boron and niobium is another possibility to increase the yield strength resulting from the steel chemical concept; all options for increasing the yield strength based on an increase in the alloying level can be characterized as passive options (∆ R_P0.2_PN_, ∆ R_P0.2_IS_, ∆ R_P0.2_SE_) aimed at improving the strength properties of the investigated material.

The maximal effect of strengthening contributions resulting from the active options obtained after cold plastic deformations ε = 50% satisfies the following sequence: ∆R_P0.2_DS_ = 560 MPa > ∆R_P0.2_RT_ =140 MPa > ∆R_P0.2_GB_ = 80 MPa.

The best values of the studied material properties were obtained after cold plastic deformations: ε = 50%: R_P0.2_ε = 50%_ = 994 MPa, A5 = 4% and d =64 μm. Meanwhile, after solution annealing, the properties were low: R_p0.2_ = 325 MPa, A5 = 49% and d = 214 μm.

The basic mechanisms of plastic deformation of austenite—Fe(γ)—with an FCC crystallographic system and a medium SFE level are deformation twinning and dislocation slips.

## Figures and Tables

**Table 1:** The formulas for the calculation of SFE [mass%].

Equation	The Authors
γSFE = 1.2+ 17.7. (%Mn) + 1.4. (%Ni) + 0.6. (%Cr) − 44.7. (%Si)	[56]
γSFE = 16.7 + 26. (%C) + 2.1. (%Ni) − 0.9. (%Cr)	[57]
γSFE = 5.53 + 17.1. (%N) + 1.4. (%Ni) − 0.16. (%Cr)	[58]
γSFE = −7.1 + 2.8. (%Ni) + 2.0. (%Mo) + 0.75. (%Mn) + 0.49. (%Cr) − 24.0. (%N) − 5.7. (%C) − 2.0. (%Si)	[59]
γSFE = 2.2 + 40. (%C) + 1.9. (%Ni) + 0.77. (%Mo) + 0.5. (%Mn) −3.6. (%N) − 2.9. (Si) − 0.016. (%Cr)	[60]

**Table 2:** The local chemical composition of 316 LN used in this paper [mass%].

C	Cr	Ni	Mn	Mo	Si	P	S	V	Ti	Nb	N	B
0.06	18.76	13.73	1.5	1.87	0.5	0.007	0.003	0.02	0.004	0.02	0.13	0.001

**Table 3:** Numerical data of DSC thermograms of samples processed by rolling at 295 K.

Treatment of Sample	Endothermic Thermal Effect					Exothermic Thermal Effect			
	Temperature [°C]					Temperature [°C]			
	T_Onset_	T_Peak_	T_Final_	Stored Energy: ΔH [J/g]	Dislocation Density: ρ [m^−2^ × 10^15^]	T_Onset_	T_Peak_	T_Final_	Stored Energy: ΔH [J/g]
After SA__peak1_	-	-	-	-	-	398	511	601	13.560
After SA__peak2_	-	-	-	-	-	759	841	843	3.691
A10__peak1_	195	274	330	1.23	1.7	-	-	-	-
A10__peak2_	-	-	-			365	433	555	2.657
A30__peak1_	164	271	376	21.28	6.8	-	-	-	-
A30__peak2_	-	-	-	-	-	463	508	537	2.427
A30__peak3_	-	-	-	-	-	795	864	-	5.649
A50__peak1_	207	277	339	1.469	5.17	-	-	-	-
A50__peak2_	-	-	-	-	-	509	569	623	2.361
A50__peak3_	-	-	-	-	-	808	877	879	7.992

**Table 4:** The calculated values of γ_SFE_ for tested material AISI 316LN with FCC crystallographic system.

Equation	Calculated Value SFE [mJ/m^2^]	Reference
γ_SFE_ = 5.53 + 17.1. (%N) + 1.4. (%Ni) − 0.16. (%Cr)	24.0	[58]
γ_SFE_ = 16.7 + 26. (%C) + 2.1. (%Ni) − 0.9. (%Cr)	30.2	[57]
γ_SFE_ = 2.2 + 40. (%C) + 1.9. (%Ni) + 0.77. (%Mo) + 0.5. (%Mn) − 3.6. (%N) − 2.9. (Si) − 0.016. (%Cr)	30.7	[60]
γ_SFE_ = 1.2 + 17.7. (%Mn) + 1.4. (%Ni) + 0.6. (%Cr)—44.7. (%Si)	35.9	[56]
γ_SFE_ = –7.1 + 2.8. (%Ni) + 2.0. (%Mo) + 0.75. (%Mn) + 0.49. (%Cr) − 24.0. (%N) − 5.7. (%C) − 2.0. (%Si)	40.9	[59]

**Table 5:** Grain boundary strengthening coefficients.

	Chemical Composition [mass %]						
Steel Grade	C	N	Cr	Ni	Mn	k_y_GB_ [MPa.μm^1/2^]	Authors
AISI 316 L	0.08	-	16.2	9.1	-	300	[85]
AISI 316 L	0.04	-	17.3	10.7	1.7	300	[86]
AISI 316 L	0.023	0.091	17.7	12.7	0.9	452	[16]
AISI 316 L	0.06	0.024	18.4	8.6	0.33	500	[87]
AISI316 LN	0.06	0.13	18.8	13.7	1.5	500	As present

**Table 6:** Individual strengthening contributions compared with literature data.

∆R_P0.2_ [MPa]	∆R_P0.2___PN_	∆R_P0.2_IS_	∆R_P0.2_SE_	∆R_P0.2_GB_	∆R_P0.2_DS_	∆R_P0.2_RT_	R_P0.2_
Min.	112	78	109	50 (ε = 0%)	103 (ε = 0%)	83 (ε = 0%)	535
Max.	123	78	126	80 (ε = 50%)	560 (ε = 50%)	140 (ε = 50%)	1107
	5–60 [25,30,31]	97 [16]	96.6 [16]	60–90 [25]	343.1 [16]		

**Table 7:** Comparison of strength property results.

	Mechanical Properties			
Processing Conditions	R_P0.2_ [MPa]	R_m_ [MPa]	A5 [%]	Reference
After annealing	≤300	550–750	>35	[32]
	325	641	49	[this article]
Cold deformation (coarse-grained structures)	≤600	-	-	[33]
	994	1057	4	[this article]
SPD techniques (UFG structures)	1100–1500	-	-	[35,36,37,38,39]

## References

[1] Fernández-Pisón P., Rodríguez-Martínez J.A., García-Tabarés E., Avilés-Santillana I., Sgobba S. (2021). Flow and Fracture of Austenitic Stainless Steels at Cryogenic Temperatures. Eng. Fract. Mech..

[2] ASM Handbook, Volume 1: Properties and Selection: Irons, Steels, and High-Performance Alloys—ASM International. https://www.asminternational.org/asm-handbook-volume-1-properties-and-selection-irons-steels-and-high-performance-alloys/results/-/journal_content/56/06181G/PUBLICATION.

[3] Singh R., Agrahari S., Yadav S.D., Kumar A. (2021). Microstructural Evolution and Mechanical Properties of 316 Austenitic Stainless Steel by CGP. Mater. Sci. Eng. A.

[4] Chen S., Sun L., Li W. (2023). Effect of Chloride Threshold on Pitting Behavior of 316LN Stainless Steel in Sour Water Solution by Electrochemical Analysis. Mater. Corros..

[5] Liu M., Cheng X., Li X., Zhou C., Tan H. (2017). Effect of Carbonation on the Electrochemical Behavior of Corrosion Resistance Low Alloy Steel Rebars in Cement Extract Solution. Constr. Build. Mater..

[6] Sonekar M.M., Rathod W.S. (2019). An Experimental Investigation on Tribologial Behavior of Bio-Implant Material (SS-316 l & Ti6Al4V) for Orthopaedic Applications. Mater. Today Proc..

[7] Xu D.M., Li G.Q., Wan X.L., Xiong R.L., Xu G., Wu K.M., Somani M.C., Misra R.D.K. (2017). Deformation Behavior of High Yield Strength—High Ductility Ultrafine-Grained 316LN Austenitic Stainless Steel. Mater. Sci. Eng. A.

[8] Czarkowski P., Krawczynska A.T., Slesinski R., Brynk T., Budniak J., Lewandowska M., Kurzydlowski K.J. (2011). Low Temperature Mechanical Properties of 316L Type Stainless Steel after Hydrostatic Extrusion. Fusion Eng. Des..

[9] Zhang H., Huang C., Huang R., Li L. (2020). Influence of Pre-Strain on Cryogenic Tensile Properties of 316LN Austenitic Stainless Steel. Cryogenics.

[10] Kvačkaj T., Bidulský R., Kováčová A., Ileninová J., Bidulská J. (2014). Analysis of Metallic Materials for Iter with the Emphasis on Copper Alloys. Acta Metall. Slovaca.

[11] Ishio K., Nakajima H. (2006). Effects of Nitrogen on the Mechanical Propeties of 316LN Stainless Steels. Tetsu-Hagane.

[12] Das A. (2019). Cyclic Plasticity Induced Transformation of Austenitic Stainless Steels. Mater. Charact..

[13] Moshtaghi M., Safyari M. (2021). Effect of Work-Hardening Mechanisms in Asymmetrically Cyclic-Loaded Austenitic Stainless Steels on Low-Cycle and High-Cycle Fatigue Behavior. Steel Res. Int..

[14] Umezawa O. (2021). Review of the Mechanical Properties of High-Strength Alloys at Cryogenic Temperatures. Mater. Perform. Charact..

[15] Wang Y., Wang Z., Wang W., Ma B. (2023). Effect of Nitrogen Content on Mechanical Properties of 316L(N) Austenitic Stainless Steel. Mater. Sci. Eng. A.

[16] Cui L., Jiang S., Xu J., Peng R.L., Mousavian R.T., Moverare J. (2021). Revealing Relationships between Microstructure and Hardening Nature of Additively Manufactured 316L Stainless Steel. Mater. Des..

[17] Sakurai T., Umezawa O. (2023). High-Strength and High-Toughness Austenitic Stainless Steels Based on Type 316LN at 4.2 K. Cryogenics.

[18] Simon N.J., Reed R.P. (1986). Strength and Toughness of Aisi 304 and 316 at 4 K. J. Nucl. Mater..

[19] Taweejun N., Kanchanomai C. (2015). Effects of Carbon and Nitrogen on the Microstructure and Mechanical Properties of Carbonitrided Low-Carbon Steel. J. Mater. Eng. Perform..

[20] Sakurai T., Iguchi M., Nakahira M. (2019). Tensile Property Evaluation of Cryogenic Structural Materials for the ITER TF Coil. Teion Kogaku.

[21] Kvackaj T., Bidulská J., Bidulský R. (2021). Overview of HSS Steel Grades Development and Study of Reheating Condition Effects on Austenite Grain Size Changes. Materials.

[22] Endoh K., Ii S., Kimura Y., Sasaki T., Goto S., Yokota T., Ohmura T. (2021). Effects of Grain Boundary Geometry and Boron Addition on the Local Mechanical Behavior of Interstitial-Free (IF) Steels. Mater. Trans..

[23] Zhai R., Zhang H., Pan S., Xu B., Liu S., Sun M. (2023). Effect of Boron Addition on the Microstructure and Cryogenic Mechanical Properties of N50 Stainless Steel after Aging Treatment. Mater. Sci. Eng. A.

[24] Behjati P., Kermanpur A., Najafizadeh A., Samaei Baghbadorani H., Karjalainen L.P., Jung J.G., Lee Y.K. (2014). Effect of Nitrogen Content on Grain Refinement and Mechanical Properties of a Reversion-Treated Ni-Free 18Cr-12Mn Austenitic Stainless Steel. Metall. Mater. Trans. A Phys. Metall. Mater. Sci..

[25] Qiu C., Kindi M.A., Aladawi A.S., Hatmi I.A. (2018). A Comprehensive Study on Microstructure and Tensile Behaviour of a Selectively Laser Melted Stainless Steel. Sci. Rep..

[26] Kvackaj T., Bidulska J. (2014). From Micro to Nano Scale Structure by Plastic Deformations. Mater. Sci. Forum.

[27] Kvackaj T., Kovacova A., Kocisko R., Bidulska J., Lityńska–Dobrzyńska L., Jenei P., Gubicza J. (2017). Microstructure Evolution and Mechanical Performance of Copper Processed by Equal Channel Angular Rolling. Mater. Charact..

[28] Šimčák D., Kvačkaj T., Kočiško R., Bidulský R., Kepič J., Puchý V. (2017). Evaluation of High Purity Aluminium after Asymmetric Rolling at Ambient and Cryogenic Temperatures. Acta Metall. Slovaca.

[29] Kováčová A., Kvačkaj T., Bidulský R., Bidulská J., Kočiško R., Dutkiewicz J., Lityńska-Dobrzyńska L. (2017). Investigation of the Ultrafine-Grained Structure Formation under Different Strain Rates. Arch. Metall. Mater..

[30] Smith T.R., Sugar J.D., San Marchi C., Schoenung J.M. (2019). Strengthening Mechanisms in Directed Energy Deposited Austenitic Stainless Steel. Acta Mater..

[31] Choudhary B.K., Christopher J. (2015). Tensile Flow and Work Hardening Behaviour of Type 316L(N) Austenitic Stainless Steel in the Framework of One-Internal-Variableand Two-Internal-Variable Approaches. Mater. Sci. Eng. A.

[32] 1.4910 (AISI 316 LN), S31653|Datasheet|METALCOR. https://www.metalcor.de/en/datenblatt/68/.

[33] Austenitic Stainless Steel: Definition, Composition, Properties, Grades, Applications and More. https://steelprogroup.com/stainless-steel/type/austenitic/#elementor-toc__heading-anchor-0.

[34] ITER—The Way to New Energy. https://www.iter.org/.

[35] Challa V.S.A., Wan X.L., Somani M.C., Karjalainen L.P., Misra R.D.K. (2014). Strain Hardening Behavior of Phase Reversion-Induced Nanograined/Ultrafine-Grained (NG/UFG) Austenitic Stainless Steel and Relationship with Grain Size and Deformation Mechanism. Mater. Sci. Eng. A.

[36] Dong F.Y., Zhang P., Pang J.C., Chen D.M., Yang K., Zhang Z.F. (2013). Optimizing Strength and Ductility of Austenitic Stainless Steels through Equal-Channel Angular Pressing and Adding Nitrogen Element. Mater. Sci. Eng. A.

[37] Zhang J., Han W., Rui W., Li J., Huang Z., Sui F. (2020). Deformation Mechanisms of 316L Austenitic Stainless Steel Tubes under Equal Channel Angular Pressing. J. Mater. Eng. Perform..

[38] Chen D., Pan Q., Liu Z., Zeng S., Shi Q., Peng J., Li Y. (2024). Microstructural Evolution, Mechanical Properties and Tribological Behavior of SiC Reinforced 316L Matrix Composites Fabricated by Additive Manufacturing. J. Mater. Res. Technol..

[39] Gubicza J., El-Tahawy M., Huang Y., Choi H., Choe H., Lábár J.L., Langdon T.G. (2016). Microstructure, Phase Composition and Hardness Evolution in 316L Stainless Steel Processed by High-Pressure Torsion. Mater. Sci. Eng. A.

[40] Üçok I., Ando T., Grant N.J. (1991). Property Enhancement in Type 316L Stainless Steel by Spray Forming. Mater. Sci. Eng. A.

[41] Xiong Y., He T., Wang J., Lu Y., Chen L., Ren F., Liu Y., Volinsky A.A. (2015). Cryorolling Effect on Microstructure and Mechanical Properties of Fe–25Cr–20Ni Austenitic Stainless Steel. Mater. Des..

[42] Roy B., Kumar R., Das J. (2015). Effect of Cryorolling on the Microstructure and Tensile Properties of Bulk Nano-Austenitic Stainless Steel. Mater. Sci. Eng. A.

[43] Xiong Y., Yue Y., Lu Y., He T., Fan M., Ren F., Cao W. (2018). Cryorolling Impacts on Microstructure and Mechanical Properties of AISI 316 LN Austenitic Stainless Steel. Mater. Sci. Eng. A.

[44] Vargas B.R.R., Albini L., Tiracorrendo G., Massi R., Stornelli G., Di Schino A. (2023). Effect of Ultrafast Heating on AISI 304 Austenitic Stainless Steel. Acta Metall. Slovaca.

[45] Kvackaj T., Rozsypalova A., Kocisko R., Bidulska J., Petrousek P., Vlado M., Pokorny I., Sas J., Weiss K.P., Duchek M. (2020). Influence of Processing Conditions on Properties of AISI 316LN Steel Grade. J. Mater. Eng. Perform..

[46] Fedoriková A., Petroušek P., Kvačkaj T., Kočiško R., Zemko M. (2023). Development of Mechanical Properties of Stainless Steel 316LN-IG after Cryo-Plastic Deformation. Materials.

[47] Lu J., Hultman L., Holmström E., Antonsson K.H., Grehk M., Li W., Vitos L., Golpayegani A. (2016). Stacking Fault Energies in Austenitic Stainless Steels. Acta Mater..

[48] Liu Y., Du J., Shang S., Zhang A., Xiong S., Liu Z.K., Liu F. (2023). Insights into Plastic Deformation Mechanisms of Austenitic Steels by Coupling Generalized Stacking Fault Energy and Semi-Discrete Variational Peierls-Nabarro Model. Prog. Nat. Sci. Mater. Int..

[49] Li W., Lu S., Hu Q.M., Johansson B., Kwon S.K., Grehk M., Johnsson J.Y., Vitos L. (2016). Generalized Stacking Fault Energy of γ-Fe. Philos. Mag..

[50] Abbasi A., Dick A., Hickel T., Neugebauer J. (2011). First-Principles Investigation of the Effect of Carbon on the Stacking Fault Energy of Fe–C Alloys. Acta Mater..

[51] Kim J.K., De Cooman B.C. (2016). Stacking Fault Energy and Deformation Mechanisms in Fe-XMn-0.6C-YAl TWIP Steel. Mater. Sci. Eng. A.

[52] Ueno H., Kakihata K., Kaneko Y., Hashimoto S., Vinogradov A. (2011). Nanostructurization Assisted by Twinning during Equal Channel Angular Pressing of Metastable 316L Stainless Steel. J. Mater. Sci..

[53] Sato A., Soma K., Mori T. (1982). Hardening Due to Pre-Existing ϵ-Martensite in an Fe-30Mn-1Si Alloy Single Crystal. Acta Metall..

[54] Seetharaman V. (1984). Deformation and Martensitic Transformation. Bull. Mater. Sci..

[55] Manjanna J., Kobayashi S., Kamada Y., Takahashi S., Kikuchi H. (2008). Martensitic Transformation in SUS 316LN Austenitic Stainless Steel at RT. J. Mater. Sci..

[56] Ojima M., Adachi Y., Tomota Y., Katada Y., Kaneko Y., Kuroda K., Saka H. (2009). Weak Beam TEM Study on Stacking Fault Energy of High Nitrogen Steels. Steel Res. Int..

[57] Brofman P.J., Ansell G.S. (1978). On the Effect of Carbon on the Stacking Fault Energy of Austenitic Stainless Steels. Metall. Trans. A.

[58] Meric de Bellefon G., van Duysen J.C., Sridharan K. (2017). Composition-Dependence of Stacking Fault Energy in Austenitic Stainless Steels through Linear Regression with Random Intercepts. J. Nucl. Mater..

[59] Rhodes C.G., Thompson A.W. (1977). The Composition Dependence of Stacking Fault Energy in Austenitic Stainless Steels. Metall. Trans. A.

[60] Yonezawa T., Suzuki K., Ooki S., Hashimoto A. (2013). The Effect of Chemical Composition and Heat Treatment Conditions on Stacking Fault Energy for Fe-Cr-Ni Austenitic Stainless Steel. Metall. Mater. Trans. A Phys. Metall. Mater. Sci..

[61] Padilha A.F., Rios P.R. (2002). Decomposition of Austenite in Austenitic Stainless Steels. ISIJ Int..

[62] El-Tahawy M., Huang Y., Choi H., Choe H., Lábár J.L., Langdon T.G., Gubicza J. (2017). High Temperature Thermal Stability of Nanocrystalline 316L Stainless Steel Processed by High-Pressure Torsion. Mater. Sci. Eng. A.

[63] Stornelli G., Albini L., Di Nunzio P.E., Tiracorrendo G., Vargas B.R.R., Di Schino A. (2023). Effect of Ultrafast Heating on AISI 441 Ferritic Stainless Steel. Acta Metall. Slovaca.

[64] Bowman A.L., Arnold G.P., Storms E.K., Nereson N.G. (1972). The Crystal Structure of Cr23C6. Acta Crystallogr. Sect. B Struct. Crystallogr. Cryst. Chem..

[65] Lai J.K.L. (1983). A Review of Precipitation Behaviour in AISI Type 316 Stainless Steel. Mater. Sci. Eng..

[66] Xu Z., Ding Z., Dong L., Liang B. (2016). Characterization of M23C6 Carbides Precipitating at Grain Boundaries in 100Mn13 Steel. Metall. Mater. Trans. A Phys. Metall. Mater. Sci..

[67] De Moraes F.P., Amaral E.M., Neto F.B., Padilha A.F., de Moraes F.P., Amaral E.M., Neto F.B., Padilha A.F. (2023). Failure Analysis of an AISI 316 Steel Pipe Elbow Exposed to the weather for Three Years after 16 Years of Operating at 515 °C. Tecnol. Metal. Mater. Min..

[68] Rollett A., Humphreys F., Rohrer G.S., Hatherly M. (2004). Recrystallization and Related Annealing Phenomena.

[69] Odnobokova M., Yanushkevich Z., Kaibyshev R., Belyakov A. (2020). On the Strength of a 316L-Type Stainless Steel Subjected to Cold or Warm Rolling Followed by Annealing. Materials.

[70] Singh K.K., Sangal S., Murty G.S. (2002). Hall–Petch Behaviour of 316L Austenitic Stainless Steel at Room Temperature. Mater. Sci. Technol..

[71] Gutierrez-Urrutia I., Zaefferer S., Raabe D. (2010). The Effect of Grain Size and Grain Orientation on Deformation Twinning in a Fe–22 Wt.% Mn–0.6 Wt.% C TWIP Steel. Mater. Sci. Eng. A.

[72] Zhu Y.T., Liao X.Z., Srinivasan S.G., Lavernia E.J. (2005). Nucleation of Deformation Twins in Nanocrystalline Face-Centered-Cubic Metals Processed by Severe Plastic Deformation. J. Appl. Phys..

[73] Zhu Y.T., Liao X.Z., Wu X.L. (2012). Deformation Twinning in Nanocrystalline Materials. Prog. Mater. Sci..

[74] Park K.T., Jin K.G., Han S.H., Hwang S.W., Choi K., Lee C.S. (2010). Stacking Fault Energy and Plastic Deformation of Fully Austenitic High Manganese Steels: Effect of Al Addition. Mater. Sci. Eng. A.

[75] Huang C.X., Wang K., Wu S.D., Zhang Z.F., Li G.Y., Li S.X. (2006). Deformation Twinning in Polycrystalline Copper at Room Temperature and Low Strain Rate. Acta Mater..

[76] Meyers M.A., Vöhringer O., Lubarda V.A. (2001). The Onset of Twinning in Metals: A Constitutive Description. Acta Mater..

[77] Fan H., Aubry S., Arsenlis A., El-Awady J.A. (2016). Grain Size Effects on Dislocation and Twinning Mediated Plasticity in Magnesium. Scr. Mater..

[78] Fan H., Aubry S., Arsenlis A., El-Awady J.A. (2015). Orientation Influence on Grain Size Effects in Ultrafine-Grained Magnesium. Scr. Mater..

[79] Kashyap K.T., Bhat A., Koppad P.G., Puneeth K.B. (2012). On Peierls Nabarro Stress in Iron. Comput. Mater. Sci..

[80] Chen S., Ma G., Wu G., Godfrey A., Huang T., Huang X. (2022). Strengthening Mechanisms in Selective Laser Melted 316L Stainless Steel. Mater. Sci. Eng. A.

[81] Pickering F.B. (1978). Physical Metallurgy and the Design of Steels.

[82] Mohd Yusuf S., Chen Y., Yang S., Gao N. (2020). Microstructural Evolution and Strengthening of Selective Laser Melted 316L Stainless Steel Processed by High-Pressure Torsion. Mater. Charact..

[83] Irvine K.J., Gladman T., Pickering F. (1969). The Strength of Austenitic Stainless Steels. J. Iron Steel Inst..

[84] Odnobokova M., Torganchuk V., Tikhonova M., Dolzhenko P., Kaibyshev R., Belyakov A. (2023). On the Strength of a 316L-Type Austenitic Stainless Steel Produced by Selective Laser Melting. Metals.

[85] Karavaeva M.V., Abramova M.M., Enikeev N.A., Raab G.I., Valiev R.Z. (2016). Superior Strength of Austenitic Steel Produced by Combined Processing, Including Equal-Channel Angular Pressing and Rolling. Metals.

[86] Belyakov A., Odnobokova M., Yanushkevich Z., Nazarova M., Kaibyshev R. (2019). On Strengthening of Ultrafine Grained Austenitic Steels Subjected to Large Strain Deformation. IOP Conf. Ser. Mater. Sci. Eng..

[87] Di Schino A., Salvatori I., Kenny J.M. (2002). Effects of Martensite Formation and Austenite Reversion on Grain Refining of AISI 304 Stainless Steel. J. Mater. Sci..

[88] Taylor G.I. (1934). The Mechanism of Plastic Deformation of Crystals. Part I.—Theoretical. Proc. R. Soc. London. Ser. A Contain. Pap. A Math. Phys. Character.

[89] Kim J.G., Enikeev N.A., Seol J.B., Abramova M.M., Karavaeva M.V., Valiev R.Z., Park C.G., Kim H.S. (2018). Superior Strength and Multiple Strengthening Mechanisms in Nanocrystalline TWIP Steel. Sci. Rep..

[90] Kim H.S., Park S.H. (2024). Significant Dislocation Strengthening of Stainless Steel 316L via Co-Directed Energy Deposition of Silica. Mater. Sci. Eng. A.

[91] Liu X., Hu R., Yang C., Luo X., Bai J., Ma R. (2023). Microstructure Evolution and Strengthening Mechanism of Γ′-Strengthening Superalloy Prepared by Laser Powder Bed Fusion. Mater. Sci. Eng. A.

[92] Khedr M., Li W., Min N., Abd-Elaziem W., Jin X. (2023). Strengthening Contributions of Mechanical Twinning and Dislocations to the Flow Stress of Hadfield High-Manganese Steel: Quantitative Analysis. J. Mater. Eng. Perform..

[93] Qin S., Lee S., Tsuchiya T., Matsuda K., Horita Z., Kocisko R., Kvackaj T. (2020). Aging Behavior of Al-Li-(Cu, Mg) Alloys Processed by Different Deformation Methods. Mater. Des..

[94] Bidulskỳ R., Bidulská J., Gobber F.S., Kvačkaj T., Petroušek P., Actis-Grande M., Weiss K.P., Manfredi D. (2020). Case Study of the Tensile Fracture Investigation of Additive Manufactured Austenitic Stainless Steels Treated at Cryogenic Conditions. Materials.

[95] Kvačkaj T., Demjan I., Bella P., Kočiško R., Petroušek P., Fedorikova A., Bidulská J., Lupták M., Jandačka P., Lascsáková M. (2022). The Influence of Annealing Temperature on the Magnetic Properties of Cryo-Rolled Non-Oriented Electrical Steel. Acta Metall. Slovaca.

[96] Petroušek P., Kvačkaj T., Bidulská J., Bidulský R., Grande M.A., Manfredi D., Weiss K.P., Kočiško R., Lupták M., Pokorný I. (2023). Investigation of the Properties of 316L Stainless Steel after AM and Heat Treatment. Materials.

[97] Bidulský R., Bidulská J., Kvackaj T., Grande M.A. (2023). Case Study of Advanced Processed OFHC Copper by Dry Sliding Wear Test. Acta Metall. Slovaca.

[98] Kapoor G., Kvackaj T., Heczel A., Bidulská J., Kociško R., Fogarassy Z., Simcak D., Gubicza J. (2020). The Influence of Severe Plastic Deformation and Subsequent Annealing on the Microstructure and Hardness of a Cu–Cr–Zr Alloy. Materials.

[99] Kvackaj T., Kočiško R., Tiža J., Bidulská J., Kovácová A., Bidulský R., Bacsó J., Vlado M. (2013). Application of Workability Test to SPD Processing. Arch. Metall. Mater..

[100] Bidulská J., Kvačkaj T., Kočiško R., Bidulský R., Actis Grande M., Donič T., Martikán M. (2010). Influence of ECAP-Back Pressure on the Porosity Distribution. Acta Phys. Pol. A.

[101] Kočiško R., Kvačkaj T., Bidulská J., Bidulský R., Petroušek P., Pokorný I., Lupták M., Actis Grande M. (2023). Evaluation of Powder Metallurgy Workpiece Prepared by Equal Channel Angular Rolling. Materials.

[102] Chung Y.H., Park J.W., Lee K.H. (2007). Controlling the Thickness Uniformity in Equal Channel Angular Rolling (ECAR). Mater. Sci. Forum.

[103] Kaščák Ľ., Varga J., Bidulská J., Bidulský R., Grande M.A. (2023). Simulation Tool for Material Behaviour Prediction in Additive Manufacturing. Acta Metall. Slovaca.

[104] Di Schino A., Stornelli G. (2022). Additive Manufacturing: A New Concept for End Users. the Case of Magnetic Materials. Acta Metall. Slovaca.

[105] Nguyen S.A., Pham K.G., Seidel C., Pham A.H., Phung C.N., Trinh T. (2023). Change in Microstructure and Hardness of Additively Manufactured AISI H13 Steel by Heat Treatment and Nitriding Processes. Acta Metall. Slovaca.

[106] Dagnaw M., Brytan Z., Bidulská J., Bidulský R. (2024). Corrosion Evaluation of LPBF-Manufactured Duplex Stainless Steel. Acta Metall. Slovaca.

